# Competitive endothelial adhesion between *Plasmodium falciparum *isolates under physiological flow conditions

**DOI:** 10.1186/1475-2875-8-214

**Published:** 2009-09-21

**Authors:** Happy Phiri, Jacqui Montgomery, Malcolm Molyneux, Alister Craig

**Affiliations:** 1 Malawi-Liverpool-Wellcome Trust Clinical Research Programme, College of Medicine, P.O. Box 30096, Chichiri, Blantyre 3, Malawi; 2 Liverpool School of Tropical Medicine, University of Liverpool, Pembroke Place, L3 5QA, Liverpool, UK

## Abstract

**Background:**

Sequestration of parasitized red blood cells in the microvasculature of major organs involves a sequence of events that is believed to contribute to the pathogenesis of severe falciparum malaria. *Plasmodium falciparum *infections are commonly composed of multiple subpopulations of parasites with varied adhesive properties. A key question is: do these subpopulations compete for adhesion to endothelium? This study investigated whether, in a laboratory model of cytoadherence, there is competition in binding to endothelium between pRBC infected with *P. falciparum *of variant adhesive phenotypes, particularly under flow conditions.

**Methods:**

Four different *P. falciparum *isolates, of known adherence phenotypes, were matched in pairs, mixed in different proportions and allowed to bind to cultured human endothelium. Using *in vitro *competitive static and flow-based adhesion assays, that allow simultaneous testing of the adhesive properties of two different parasite lines, adherence levels of paired *P. falciparum *isolates were quantified and analysed using either non-parametric Wilcoxon's paired signed rank test or Student paired test.

**Results:**

Study findings show that *P. falciparum *parasite lines show marked differences in the efficiency of adhesion to endothelium.

**Conclusion:**

*Plasmodium falciparum *variants will compete for adhesion to endothelia and variants can be ranked by their efficiency of binding. These findings suggest that variants from a mixed infection will not show uniform cytoadherence and so may vary in their ability to cause disease.

## Background

The pathogenicity of *Plasmodium falciparum *is thought to result in part from the unique ability of *P. falciparum*-infected erythrocytes (pRBC) to adhere to, and activate, vascular endothelium. The primary process of cytoadherence has been studied in detail and is mediated by a variety of host endothelial receptors and *P. falciparum *antigens expressed on the surface of pRBC. *Plasmodium falciparum *erythrocyte membrane protein 1 (PfEMP1) is a major variant surface antigen expressed on the surface of pRBC that mediates cytoadherence through its interaction with a diverse array of receptors that are expressed on the surface of vascular endothelial cells, infected and uninfected erythrocytes and platelets [1,2]. Several host receptors of clinical interest involved in this process have been identified and described in detail [3], including intercellular adhesion molecule-1 (ICAM-1) [4] and CD36 [5,6].

Previous studies comparing *P. falciparum *isolates have demonstrated differential parasite binding to endothelial cells and also to purified receptors [7,8], including ICAM-1, which has allowed categorization of *P. falciparum *isolates into low- and high-ICAM-1-avidity binders [7]. A range of primary endothelial cell lines have been derived from different tissues and can be used as laboratory models to study cytoadherence. Examples include macrovascular human umbilical vein endothelial cells (HUVEC) and dermal microvascular endothelium (HDMEC). HDMEC constitutively express CD36 and low levels of ICAM-1, and can also be induced to express high levels of ICAM-1, vascular cell adhesion molecule 1 (VCAM-1) and P-selectin using agonists such as tumour necrosis factor (TNF) [9,10]. In contrast, HUVEC are CD36-deficient but constitutively express small amounts of ICAM-1, which is up-regulated on stimulation by TNF [8,11,12].

A previous study characterising binding of four laboratory isolates (JDP8, ItG, A4 and C24) to purified receptors (ICAM-1 and CD36) and endothelial cells (HUVEC and HDMEC), under both static and flow conditions, showed a range of binding capabilities [8]. The molecular basis for this difference is not known but could be due to variation in the binding sites for major receptors, such as those seen in ICAM-1 [13] as well as differences in the display and copy number of parasite adhesins on the surface of the infected red blood cell, such as seen in HbC [14]. Previous studies [8,15,16] have suggested that this disparity in adhesion could also be due to differences in the length of PfEMP1 protein, which has implications for the accessibility and mobility of the molecule under flow conditions. For example, the PfEMP1 molecules expressed by ItG and JDP8 are considerably shorter than those expressed by A4 [8] and this could compromise the efficiency of tethering under flow.

The presence of more than one parasite line (genetically or phenotypically mixed infection) is a common feature of natural infections, particularly in malaria endemic areas [17]. However, this raises the question of whether parasite variants have equal access to different endothelia, or if certain variants out-compete others for adhesion in specific vascular sites. In this study we investigated whether competition (based on the efficiency of adhesion) between pRBC takes place on endothelium, particularly under flow conditions which mimic more closely the situation *in vivo*. To address this question, different laboratory *P. falciparum *strains were used to examine their ability to bind to human endothelial cells under both static and flow conditions. Competition was defined as an alteration in the relative ability of single *P. falciparum *parasite lines to bind endothelia, when two lines are mixed in a single experiment.

## Methods

### Malaria parasites

Four *Plasmodium falciparum *lines, C24 [8,18], A4 [8,18], ItG [8,19] and JDP8 [8,20], were used. These laboratory-adapted parasite lines have been independently tested for binding to both HUVEC and HDMEC under both static and flow conditions in our laboratory [8], so their binding abilities to the two endothelia are known. The characteristics of these parasite lines are summarized in Table 1. Parasite lines ItG, A4 and C24 are from the IT lineage. JDP8 is a culture-adapted patient isolate from Madhya Pradesh, India.

**Table 1:** Adhesion of C24, A4, ItG and JDP8 pRBC per mm^2 ^to purified receptors and endothelial cells as shown by Gray *et al*., 2003.

	**ICAM-1** **(100 μg/ml)**	**CD36** **(2 μg/ml)**	**HUVEC** **(ICAM-1)**	**HDMEC** **(ICAM-1, CD36)**
C24	291 ± 85	2797 ± 740	19 ± 4	73 ± 14
A4	2082 ± 421	1655 ± 408	37 ± 2	707 ± 57
ItG	5615 ± 738	2589 ± 326	224 ± 12	1521 ± 124
JDP8	6701 ± 1148	407 ± 122	231 ± 23	1134 ± 65

This data describes the binding phenotypes of four laboratory isolates, used in the present study, when independently tested under static conditions. Static assays were carried out as described previously [4,8]. Data shown is the mean number of pRBC per mm^2 ^± standard error of the means of 3-5 experiments.

### Preparation of parasites for adhesion assays

For each adhesion assay, the two parasite lines to be compared were cultured using a modified version of a method described previously [21,22]. Parasites were used at similar trophozoite stages at 5% parasitaemia and 2% haematocrit. Prior to the assays, two parasite lines were mixed in five different proportions of 100:0, 80:20, 50:50, 20:80 and 0:100 in order to artificially create heterogeneous parasite populations. Prior to mixing, one of the parasite suspensions was labelled with ethidium bromide (EtBr) as described previously [23]. In repeat experiments, the alternate parasite line was labelled. Cooke *et al *compared five different fluorochromes for their specificity of staining, intensity of fluorescence and effect on adhesive properties of pRBC, and EtBr was chosen as it fluoresces intensely, does not stain uninfected erythrocytes and does not leak out of the cells within the timeframe of the assay [23]. EtBr is excitable by green light illumination (510-560 nm), and gives less rapid quenching of the fluorescent signal than ultraviolet light, allowing ready detection throughout the experimental procedure [24]. For flow-based adhesion assays, the mixed suspensions were kept in the dark at 37°C until required, for a maximum of 10 min.

### Static adhesion assays

Static adhesion assays were carried out using a modified version of a previously described method [4]. Briefly, HUVEC or HDMEC (third passage) were seeded onto 1% gelatin-coated 13 mm Thermanox coverslips (Nalgene, Nunc). Once confluent, they were incubated overnight at 37°C with 1 ng/ml recombinant TNF (Biosource International). Cells were washed with binding buffer (RPMI 1640, supplemented with 6 mM glucose, pH 7.2) and incubated with 0.5 ml parasite suspension for 1 hr at 37°C with gentle resuspensions every 10 min. Unbound parasites were removed by a 1 hr gravity wash. Levels of adhesion were quantified by direct microscopic examination at ×300 magnification. The numbers of adherent pRBC of both parasite lines were counted in three separate areas of the coverslip. As one of the parasite lines in the mixed parasite suspension was stained with EtBr, the two variant parasite populations could be defined separately by viewing under white, followed by green, illumination.

### Competitive flow adhesion assays

Flow adhesion assays were carried out using a modified version of a previously described method [8]. Briefly, endothelial cells were grown in microslides for up to 48 hrs. Upon reaching confluence, endothelia were incubated overnight at 37°C with 1 ng/ml TNF, using intermittent flow to exchange the tissue culture medium in the microslides. Two parasite lines, mixed in the five different proportions, were flowed through the microslide and allowed to adhere to endothelial cells on the lower internal surface of the microslide at a wall shear stress of 0.05 Pa for a total of 5 min. Binding buffer was then flowed through at the same rate for 5 min to remove non-adherent pRBC. All assays were performed at 37°C, and the wall shear stress mimics shear stress in post-capillary venules where adhesion occurs *in vivo *[25]. As above, one of the parasite lines was stained with EtBr for identification purposes. The number of stationary pRBC in a single field of view was counted under each illumination at ×300 magnification. For each experiment, the numbers of stationary pRBC were counted in 5 separate microscopic fields.

### Data management and statistical analysis

The number of adherent pRBC was expressed per mm^2^. Experiments performed on different days showed a wide variation in the absolute number of adherent pRBC. To standardize between experiments, the number of adherent pRBC/mm^2 ^from the paired parasite lines was further expressed as a percentage ratio such that in each field of view counted, the sum of the proportions of adherent pRBC of the two parasite lines was equal to 100%. To compare levels of adhesion between paired parasite lines, the ratios of adherent pRBC at 80:20, 50:50 and 20:80 for each parasite line were expressed as mean ratios of all the experiments performed. Thus, the data comparing levels of adhesion between paired parasite lines is shown as mean ratio ± standard error of the means of all experiments performed.

As the number of paired observations was <10, the non-parametric Wilcoxon's paired signed rank test was used to measure differences in adhesion between paired parasite lines in each static assay. For all flow assays, comparisons between the paired parasite lines were tested using the Student's paired t-test, except where the number of paired observations was <10, in which case Wilcoxon's paired signed rank test was used. Tests were performed using the ratio of adherent parasites/mm^2 ^for each parasite line and p-values were obtained for each comparison made. Calculations were performed using the SPSS 11.0 statistical package.

## Results

Competitive static and flow adhesion assays were performed to compare the level of adhesion between competing parasite lines. Similarly to previous studies [23], >98% of adherent cells in all assays were pRBC.

### Static adhesion to HUVEC

**Figure 1:**
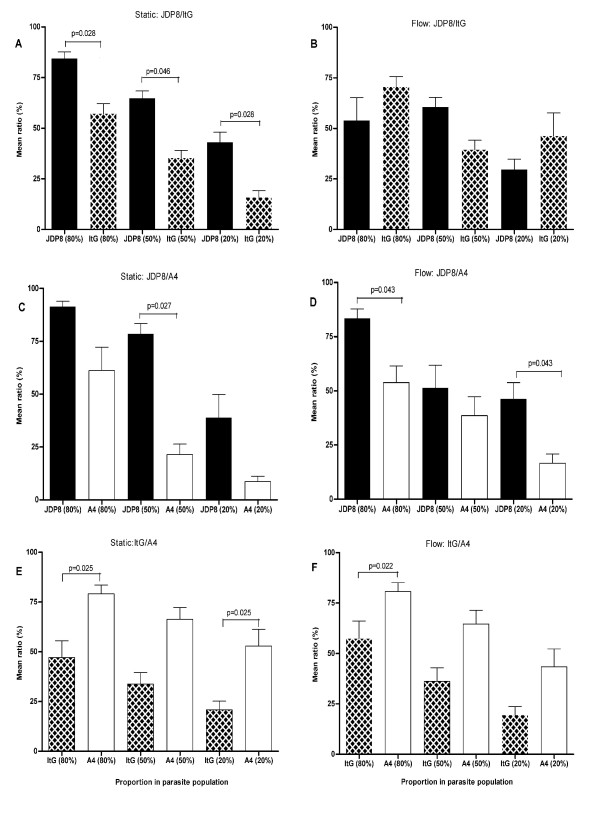
**Adhesion of JDP8, ItG and A4 to HUVEC under both static and flow conditions**. Data is shown as mean ratio of binding for each pRBC pair ± standard error of the means of 2-3 experiments. Parasite lines are plotted adjacently by proportion in each experiment, rather than as paired in the adhesion assays, for ease of statistical interpretation. Differences between mean ratios of paired parasite lines were statistically significant if P < 0.05.

When mixed JDP8 and ItG populations were exposed to TNF-activated HUVEC, JDP8 adhesion levels (in terms of relative binding proportion rather than absolute amounts) were significantly greater than ItG, regardless of the initial proportion of parasites in the population (p = 0.028, p = 0.046 and p = 0.028 for proportions JDP8:ItG of 80:20, 50:50 and 20:80 respectively; Figure 1A) and in contrast to single population assays where the parasites bind with equal avidity. This was particularly noticeable for the 20:80 mix where JDP8 adhesion was responsible for over 40% of the binding (p < 0.028). Similarly, assays using a mixture of JDP8 and A4 showed that JDP8 adhesion levels were consistently higher than A4. However, the difference was only significant when the two parasite lines were mixed equally (p = 0.027; Figure 1C). Contrary to previous studies in our laboratory with pure suspensions [8], A4 showed significantly greater adhesion levels than ItG when mixed in the proportions of 80:20 and 20:80 (p = 0.025; Figure 1E), but just failed to reach significance when mixed 50:50 (p = 0.058).

### Static adhesion to HDMEC

**Figure 2:**
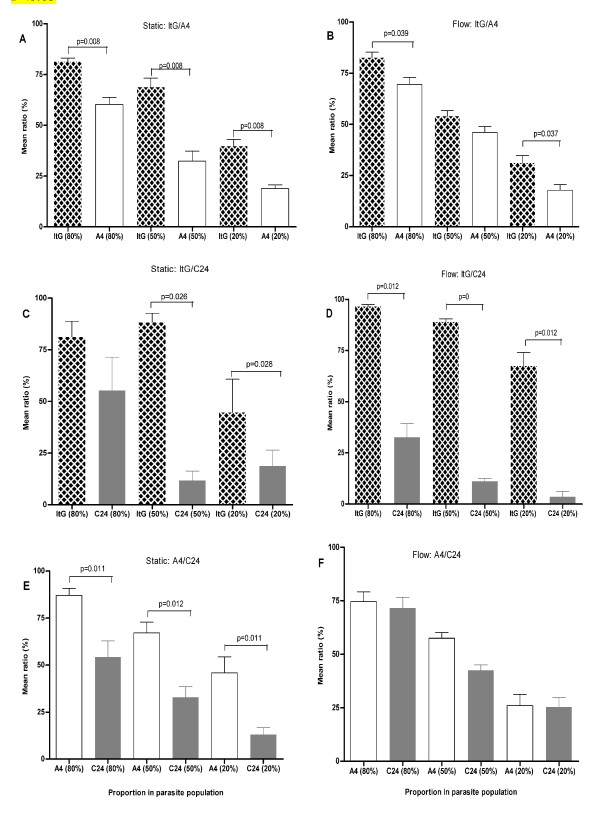
**Adhesion of ItG, A4 and C24 to HDMEC under both static and flow conditions**. Data is shown as mean ratio of binding for each pRBC pair ± standard error of the means of 2-3 experiments. Parasite lines are plotted adjacently by proportion in each experiment, rather than as paired in the adhesion assays, for ease of statistical interpretation. Differences between mean ratios of paired parasite lines were statistically significant if P < 0.05.

In contrast to HUVEC, ItG adhesion to TNF-activated HDMEC was significantly higher than that of A4, regardless of the initial proportion of ItG parasites in the mixed parasite population (p = 0.008 for all proportions; Figure 2A), indicating an important role for CD36 in this iteration. Similarly, and also in agreement with previous studies with pure suspensions [8] (Table 1), A4 showed significantly higher levels of adhesion than C24, irrespective of their proportions in the mixtures (p = 0.011 for both 80:20 and 20:80, and p = 0.012 for 50:50; Figure 2E). In a mixed suspension of ItG and C24, it was found that ItG demonstrated significantly greater levels of adhesion than C24 when mixed in 50:50 (p = 0.026; Figure 2C) and 20:80 (p = 0.028) proportions but not when ItG was the main component of the population (p = 0.344).

### Competitive adhesion under flow to HUVEC

Contrary to previous findings using pure suspensions [8], JDP8 adhesion to activated HUVEC was significantly higher than A4 when mixed in the proportions of 80:20 and 20:80 under flow conditions (p = 0.043, for both; Figure 1D). Similarly, JDP8 demonstrated greater levels of adhesion than ItG when mixed in 50:50 proportions albeit the difference between the two parasite lines was not statistically significant (p = 0.08; Figure 1B). Surprisingly, when these parasite lines were mixed in the proportions of 80:20 or 20:80 under flow conditions, ItG demonstrated greater levels of adhesion than JDP8 (Figure 1B), although again these differences were not statistically significant.

When ItG and A4 were mixed in the proportion of 80:20 and exposed to TNF-activated HUVEC, A4 showed significantly greater levels of binding under flow conditions than ItG (p = 0.022; Figure 1F), but just failed to reach significance when mixed in equal proportions (p = 0.054). This is in agreement with previous studies in our laboratory using pure suspensions [8] where a similar trend was observed. A4 adhesion was responsible for more than 80% of binding when A4 was the main component of the population.

### Competitive adhesion under flow to HDMEC

Using a mixture of ItG and C24, it was found that ItG bound strongly to activated HDMEC, and while C24 also bound HDMEC, levels of adhesion were significantly lower in all the mixes (p = 0.012, p = 0.000 and p = 0.012 for proportions 80:20, 50:50 and 20:80 respectively; Figure 2D). This was in line with a previous study using a pure suspension of C24, where binding of C24 under flow conditions was minimal [8]. Similarly, ItG adhesion to activated HDMEC under flow conditions was consistently higher than A4, irrespective of their proportions in the population, although this was not statistically significant when ItG and A4 were mixed in equal proportions (p = 0.174; Figure 2B).

Assays using a mixture of A4 and C24 showed that C24 adhesion to HDMEC was minimal (Figure 2F), a trend observed in previous single population assays [8]. However, the difference between the two parasite lines was not statistically significant for any mixture (p = 0.684, p = 0.102 and p = 1.0 for proportions 80:20, 50:50 and 20:80 respectively).

## Discussion

Matched pairs of four phenotypically-different parasite lines have been used to investigate competition in terms of their efficiency to bind to human tissue-derived endothelial cells using *in vitro *static and flow-based adhesion assays that allow simultaneous testing of the adhesive properties of two different parasite lines. Binding studies using purified receptors ICAM-1 and CD36 have shown that ItG is a strong ICAM-1 binder that also adheres to CD36 [19]. A4 binds to purified ICAM-1 moderately and to CD36 strongly [26], and C24 only binds CD36 [26]. JDP8 binds ICAM-1 with similar avidity to ItG but has relatively low binding to CD36 [8,20] (Table 1).

In mixed suspensions, we demonstrate that under static conditions, JDP8 binds more efficiently to TNF-activated HUVEC than either ItG or A4. This finding is in agreement with previous data showing that strong ICAM-1 binders are more efficient at adhering to activated endothelial cells [8]. Comparison between ItG and A4 adhesion to HUVEC under static conditions gave unexpected results, considering that ItG was previously shown to be a stronger ICAM-1 binder than A4. The parasite line A4 clearly demonstrated greater levels of adhesion to HUVEC than ItG at all proportions. Gray *et al *[8] reported that the expressed PfEMP1 molecules on ItG and JDP8 are considerably shorter than those expressed on A4, and because PfEMP1 mediates parasite binding to endothelium, this has implications for the accessibility and mobility of the molecule under flow conditions. It is possible that the shorter PfEMP1 molecules on ItG may affect bond rigidity as well as the efficiency of making contact with expressed ICAM-1 under flow. This may occur by reducing the flexibility and accessibility of the molecule, ultimately reducing the efficiency of initiation of binding [15,20]. This may explain the difference between ItG and A4 binding to HUVEC under flow conditions, seen both in the present and in previous studies. While differences in the length of expressed PfEMP1 molecules provide a credible explanation as to why A4 binds HUVEC more strongly than ItG under flow conditions, this does not explain why A4 binds better than ItG under static conditions when presented in a mixture of variant types. Likewise, differential parasite avidity and/or affinity for the ICAM-1 expressed on HUVEC does not explain this finding and further work to investigate the interaction with ICAM-1 or the role of other endothelial receptors is warranted.

In contrast to static adhesion to HUVEC, ItG adhesion to activated HDMEC was significantly higher than A4, regardless of the initial proportion of ItG parasites in the mixed parasite population. This finding can be ascribed to differences in their binding avidities to the expressed ICAM-1 and CD36 on HDMEC. The availability of both CD36 and ICAM-1 expressed by HDMEC at high levels gives ItG an advantage to bind more strongly than A4 considering that ItG binds ICAM-1 strongly but also adheres relatively strongly to CD36, while A4 binds strongly to CD36, but has relatively weaker binding to ICAM-1.

Similarly to static assays, ItG also demonstrated greater adhesion levels to activated HDMEC than A4 under flow conditions. This finding confirms earlier observations that ICAM-1 plays a major role in mediating pRBC adhesion under flow conditions [8]. Gray *et al *demonstrated that elevated *P. falciparum *adhesion to HDMEC requires capture of pRBC from flow, and this is highlighted by the ability of ItG (a strong ICAM-1 binder) to bind HDMEC better than A4 (a weaker ICAM-1 binder) in the present study. This may also explain why both ItG and A4 bound HDMEC significantly higher than C24 as previous studies [27,28,8] have demonstrated that ICAM-1 and CD36 cooperate to mediate efficient pRBC cytoadherence.

Published work has shown that although ItG and JDP8 bind ICAM-1 efficiently, these two parasite lines adhered poorly to TNF-activated HUVEC under flow conditions despite the fact that activated HUVEC expresses high levels of ICAM-1. In contrast, the parasite line A4, which is a weaker ICAM-1 binder under static conditions on both immobilized ICAM-1 and HUVEC, demonstrated much greater levels of binding to HUVEC under flow conditions. This raises the question of how parasite lines with ItG- and JDP8-like adhesive behaviour will bind to cerebral endothelium, considering that the pattern of receptor expression on HUVEC is similar to that on brain endothelium with both lacking significant amounts of CD36, unless supplemented by the action of platelets [29,30]. Competitive flow-based adhesion assays using JDP8/A4 and ItG/A4 gave conflicting results. Although a comparison of competitive binding to activated HUVEC between JDP8 and A4 appears to support previous findings [8], when A4 was compared with another strong ICAM-1 binder, ItG, the results were inconsistent with previous work. Why might this be?

**Figure 3:**
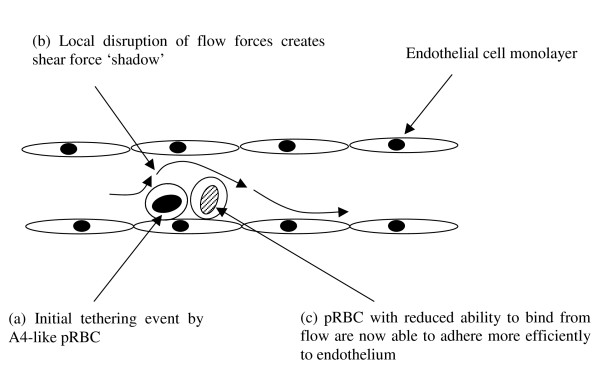
**Schematic diagram illustrating the hypothesis of amplification of pRBC**. sequestration by pRBC-induced local mechanical disruption of flow (Diagram adapted from Chakravorty and Craig, 2005). The initial binding of one parasite line may create an obstruction for the blood flow thus allowing another parasite line, which requires slower flow rates for efficient binding, to adhere.

One hypothesis to explain these discrepancies is that the initial binding of one parasite line creates an obstruction for the blood flow, generating a shear stress shadow that may enable other parasites that require a slower flow rate to adhere *in vivo *(Figure 3). While cytoadherence in microvessels was mimicked in our flow model, this model does not produce a large flow shadow, but other studies by us (unpublished observations) have suggested highly localized alterations in flow caused by obstruction on the flow plate, such as adherent cells. Shelby *et al*. (2003) using fabricated microchannels to mimic capillaries of different dimensions showed that early- and late-stage *P. falciparum *trophozoites had difficulties passing through 2-4 μm channels but that they could traverse channels of 6-8 μm in diameter [31]. The present study used microslides (300 μm in cross-section) which are much wider. Thus, as well as inherent differences in efficiencies of adhesion between different variants, the physical interaction within capillaries might also need to be taken into consideration. Future competition or interaction studies should consider using artificial vessels with appropriate dimensions in order to accurately study the behaviour of pRBC under capillary-like conditions. Other options however are that A4 is more efficient in its adhesion to activated HUVEC than ItG or that the presence of CD36 on HDMEC combines with the action of ICAM-1 to produce different effects on the efficiency of adhesion of the parasite variants, which could have implications for clinical correlation studies using single receptor targets.

This study set out to identify differences in parasite adhesion between different *P. falciparum *antigenic variants when mixed and exposed to endothelial cells simultaneously. Given the density of endothelial cell receptors available in this assay system, it is unlikely that these are limiting and so the main variable being measured is the relative efficiency of adhesion between the two isolates being tested. This study demonstrates that *P. falciparum *parasite lines show marked differences in the efficiency of adhesion to endothelium, which is not new, but more importantly that findings from assays using single receptors and parasite lines may not provide a full picture as to the situation *in vivo*. The findings suggest that variants in a mixed infection will potentially show a spectrum of cytoadherence behaviour and so despite a mixture of parasite types being present in the circulation, specific variants may vary in their efficiency of binding to particular microvascular beds as well as in their pathogenicity. This is in agreement with a recent study on the dynamics of *var *expression in tissues of fatal paediatric malaria patients that demonstrated tissue-specific accumulation of variant types of parasites [32]. Tissue-specific retention of particular parasites is presumably a consequence of favourable host-parasite binding interactions.

## Conclusion

The present study clearly shows that *P. falciparum *variants will compete for adhesion to endothelia based on their efficiency of binding. This suggests that variants from a mixed infection will not display uniform cytoadherence and so may vary in their ability to cause disease.

Finally, the competitive *in vitro *static and flow-based adhesion assays allow us to rank *P. falciparum *variants in terms of their efficiency of binding (Table 2). The term 'efficiency' has been used widely in describing cytoadherence and it has been assumed that 'efficient' binders will cause severe disease. However, almost no data to support these statements has been presented. Future studies should consider investigating relative binding efficiencies of clinical *P. falciparum *isolates and their association with disease. Such studies will certainly further understanding of cytoadherence and sequestration, and how these two processes contribute to pathogenesis of disease.

**Table 2:** ItG, JDP8, A4 and C24 ranked according to their binding efficiencies to HUVEC and HDMEC in competitive static and flow adhesion.

**Rank**	**Static assay**		**Flow assay**	
		
	**HUVEC**	**HDMEC**	**HUVEC**	**HDMEC**
1	JDP8	ItG	JDP8	ItG
2	A4	A4	A4	A4
3	ItG	C24	ItG	C24

## Competing interests

The authors declare that they have no competing interests.

## Authors' contributions

HP performed laboratory work, participated in study design and drafted the manuscript. AC conceived of the study, participated in its design and helped to draft the manuscript. JM and MM assisted with drafting the manuscript. All authors read and approved the final manuscript. All authors contributed significantly to this work and declare no conflict of interest.
